# Are complications related to the perineal post on orthopaedic traction tables for surgical fracture fixation more common than we think? A systematic review

**DOI:** 10.1186/s13037-023-00355-y

**Published:** 2023-03-22

**Authors:** Andrea Attenasio, Matthew J. Kraeutler, Ian S. Hong, Suriya Baskar, Deepak V. Patel, Craig Wright, Jaclyn M. Jankowski, Frank A. Liporace, Richard S. Yoon

**Affiliations:** 1 Division of Orthopaedic Trauma & Adult Reconstruction, Department of Orthopaedic Surgery, Cooperman Barnabas Medical Center/Jersey City Medical Center – RWJBarnabas Health, 377 Jersey Ave, Suite 550, Livingston, Jersey City, NJ 07302 USA; 2 Department of Orthopedics & Sports Medicine, Houston Methodist Hospital, Houston, TX USA; 3 Department of Orthopaedic Surgery, St. Joseph’s University Medical Center, Paterson, NJ USA

**Keywords:** Femur fracture, Perineal post, Complications, Pudendal neurapraxia, Traction table

## Abstract

**Background:**

Traction tables have long been utilized in the management of fractures by orthopaedic surgeons. The purpose of this study was to systematically review the literature to determine the complications inherent to the use of a perineal post when treating femur fractures using a traction table.

**Methods:**

A systematic review was conducted using PRISMA (Preferred Reporting Items for Systematic Reviews and Meta-Analyses) using PubMed, EMBASE, and Cochrane Library. The search phrase used was “fracture” AND “perineal” AND “post” AND (“femur” OR “femoral” OR “intertrochanteric” OR “subtrochanteric”). Inclusion criteria for this review were: level of evidence (LOE) of I – IV, studies reporting on patients surgically treated for femur fractures, studies reporting on patients treated on a fracture table with a perineal post, and studies that reported the presence or absence of perineal post-related complications. The rate and duration of pudendal nerve palsy were analyzed.

**Results:**

Ten studies (2 prospective and 8 retrospective studies; 2 LOE III and 8 LOE IV) were included consisting of 351 patients of which 293 (83.5%) were femoral shaft fractures and 58 (16.5%) were hip fractures. Complications associated with pudendal nerve palsies were reported in 8 studies and the mean duration of symptoms ranged between 10 and 639 days. Three studies reported a total of 11 patients (3.0%) with perineal soft tissue injury including 8 patients with scrotal necrosis and 3 patients with vulvar necrosis. All patients that developed perineal skin necrosis healed through secondary intention. No permanent complications relating to pudendal neurapraxia or soft tissue injuries were reported at final follow-up timepoints.

**Conclusion:**

The use of a perineal post when treating femur fractures on a fracture table poses risks for pudendal neurapraxia and perineal soft tissue injury. Post padding is mandatory and supplemental padding may also be required. Appropriate perineal skin examination prior to use is also important. Occurring at a higher rate than previously thought, appropriate post-operative examination for any genitoperineal soft tissue complications and sensory disturbances should not be ignored.

## Introduction

The application of traction in fracture reduction is an extensively studied and practiced facet of orthopaedics [1]. Traction tables have long been utilized in the management of fractures by orthopaedic surgeons [2]. Presently, the traction table is used prominently in hip arthroscopy [3, 4] and anterior total hip arthroplasty [5, 6]. While traction tables are still being used for femur fractures, comparative studies evaluating the use of traction table versus manual traction have reported results in favor of the latter due to reduced operative times [7, 8]. A recent survey of patient positioning preferences for femoral intramedullary nailing by Rubinger et al. [9] found that only 27% of American surgeon respondents preferred using traction table compared to 89% of the Canadian surgeons. As beneficial as these tables have been, they are not without their own drawbacks and complications.

Reported adverse events of fracture table utilization include fracture malrotation [5], fracture malalignment [10, 11], neurologic injury (sciatic, common peroneal, pudendal) [12–18], and soft tissue injury [19–21]. Many of these complications are a result of the use of a perineal post that functions as the point of countertraction or due to traction forces applied intraoperatively. Studies which have evaluated the mechanisms behind these complications indicate that the traction force and time under traction are important risk factors for groin-related complications [22, 23]. Pudendal nerve palsy seems to be the most common complication as the nerve becomes vulnerable to compression between the post and the ischium. In 2010, Flierl et al. [24] published a narrative literature review, which presented a comprehensive overview and expert-analysis of traction table-related complications in various orthopaedic procedures including hip arthroscopy, minimally invasive total hip replacements, trauma, and femoral fracture fixation. The authors provided evidence-based recommendations, such as the use of a radiolucent standard operating table for obese patients, optimizing patient positioning, ensuring adequate padding of the perineal post, and reducing operating time when feasible, to mitigate these devastating complications.

Although there may be a trend away from the use of traction tables for femoral fracture management, it is not uncommonly used. Thus, it is imperative to identify and analyze these perineal post-related complications to make surgeons aware of the risks and influence a change in management practices or develop effective countermeasures to implement in the operating room. The purpose of this study was to systematically review the literature to determine the complications inherent to the use of a perineal post in the treatment of femur fractures.

## Methods

### Search strategy

A comprehensive literature search was performed on May 31, 2022 by ISH using PubMed, EMBASE, and Cochrane Library databases of all available literature at the time of search. Guidelines outlined in the Preferred Reporting Items for Systematic Reviews and Meta-Analyses (PRISMA) were followed [25].Using Boolean operators a medical subject headings (MeSH) term was generated that was used for each database: “fracture” AND “perineal” AND “post” AND (“femur” OR “femoral” OR “intertrochanteric” OR “subtrochanteric”). Inclusion criteria for this review were: (1) original studies, (2) level of evidence of I – IV, (3) studies reporting on hip fracture patients of all ages treated on fracture table with a perineal post, (4) studies that reported the presence or absence of perineal post-related complications, (5) literature with the primary language in English, and (6) all literature available within the database with no restrictions on year of publication. Exclusion criteria included: (1) conference abstracts, (2) case reports, (3) biomechanical studies, (4) cadaveric studies, (5) editorial commentaries, (6) technique articles, (7) review articles, (8) expert opinion, (9) articles not written in English, (10) articles that did not report complications relating to the perineal post, and (11) articles that reported surgical management of injuries other than femur fractures. 

### Study selection

Covidence systematic review software (Veritas Health Innovation, Melbourne, Australia available at www.covidence.org), a web-based collaboration software program that streamlines the production of systematic and other literature reviews was utilized for screening titles and abstracts and subsequently the full-length articles. The full-length articles were accessed and uploaded onto Covidence by ISH. Two independent reviewers (ISH and AA) reviewed studies for eligibility criteria using the predetermined inclusion and exclusion criteria. A third author (MJK) was consulted for the final decision when there was disagreement between the two independent reviewers to mediate the process of study selection. Inter-rater reliability (IRR) for full-text screening can be found in (Table 1).

**Table 1:** Inter-Rater Reliability for Full-Text Screening

**Reviewer decisions**	
*1 = include, 2 = include*	7
*1 = include, 2 = exclude*	0
*1 = exclude, 2 = include*	0
*1 = exclude, 2 = exclude*	3
**Proportionate agreement**	1
**Yes probability**	0.49
**No probability**	0.09
**Random agreement probability**	0.58
**Cohen’s Kappa**	1

Reviewer 1 = author AA, Reviewer 2 = author ISH

### Data extraction

After the full-text screening phase of PRISMA guidelines, data from studies that were deemed eligible for inclusion in this review were extracted and inputted into a spreadsheet database created by ISH. Data that were extracted included: article title, first author name, journal, publication year, study design, level of evidence, sample size, patient demographic data (sex, mean age at time of surgery, mean time from injury to surgery, mechanism of injury, injury characteristics, body mass index [BMI], and co-morbidities), operative data (mean operating time, position on fracture table, fracture fixation method, manufacturer of fracture table, details and dimensions of the perineal post used), postoperative complications related to perineal post (rate and duration of pudendal nerve palsy; erectile dysfunction [ED]; unilateral sensory disturbance of labia, scrotum or penis; peroneal palsy, perineal skin necrosis, testicular swelling and scrotal edema, urinary retention), and only 1 patient-reported outcome score (PRO). The international index of erectile function (IIEF) which is a multi-dimensional self-reported PRO for evaluating sexual function and severity of ED in males. The IIEF score measures five domains of male sexual function within the past 4 weeks and includes erectile function, orgasmic function, sexual desire, intercourse satisfaction, and overall satisfaction.

### Appraisal of quality of study methodology and risk of bias

The methodologic index for non-randomized studies (MINORS) was used to characterize the methodological quality and risk of bias for all studies that were included in final review (Table 2) [26]. Analysis of the mean ± SD global scores for comparative (total global score of 24) and noncomparative (total global score of 16) studies was performed. Higher MINORS scores reflect a higher quality study methodology and correlates to a lower risk of bias.

**Table 2:** Methodologic index for non-randomized studies

Study	Clearly stated aim	Inclusion of consecutive patients	Prospective collection of data	Endpoints appropriate to the aim of the study	Unbiased assessment of the study endpoint	Follow-up period appropriate to the aim of the study	Loss to follow-up less than 5%	Prospective calculation of the study size	Additional criteria in the case of comparative study				Overall Score
First author (year)									Adequate control group	Contemporary groups	Baseline equivalence of groups	Adequate statistical analyses	
**Aprato et al. (2021)**	2	2	0	2	0	1	2	0	2	1	2	2	**16/24**
**Brumback et al. (1992)**	2	2	2	2	2	1	1	0	2	2	0	1	**17/24**
**Kao et al. (1993)**	1	0	0	2	0	1	2	0	–	–	–	–	**6/16**
**Mallet et al. (2005)**	2	2	0	2	0	2	1	0	2	2	2	2	**17/24**
**Parulekar et al. (2021)**	0	2	0	2	0	2	2	0	–	–	–	–	**8/16**
**Peterson et al. (1985)**	0	2	0	2	0	2	0	0	–	–	–	–	**8/16**
**Rajbabu et al. (2007)**	0	2	0	2	0	2	2	0	–	–	–	–	**8/16**
**Coelho et al. (2008)**	2	2	0	2	0	2	2	0	–	–	–	–	**10/16**
**Rose et al. (2007)**	2	2	2	2	0	0	2	0	–	–	–	–	**10/16**
**Hofmann et al. (1982)**	2	0	0	2	0	1	2	0	–	–	–	–	**7/16**
**Summary Statistics**									**Mean ± SD (range) global score for 3 comparative studies = 16.7 ± 0.6 (16– 17)**				**Mean ± SD global score for 7 noncomparative studies = 8.1 ± 1.5 (6–10)**

The items on the MINORS are scored 0 (not reported), 1 (reported but inadequate) or 2 (reported and adequate). The maximum global score is 16 for noncomparative studies and 24 for comparative studies. “–“ denotes MINORS criteria that was not applicable for appraisal

### Statistical analysis

All statistical analyses were performed used SPSS version 25 (IBM Corporation, Armonk, New York). Standard descriptive statistics were reported including measures of central tendency, variability as well as frequencies and proportions. Inter-rater reliabilities of the two independent reviewers during PRISMA screening were assessed using Cohen’s kappa and the joint probability of agreement that could be expected to occur through chance was reported.

## Results

### Study selection

**Fig. 1:**
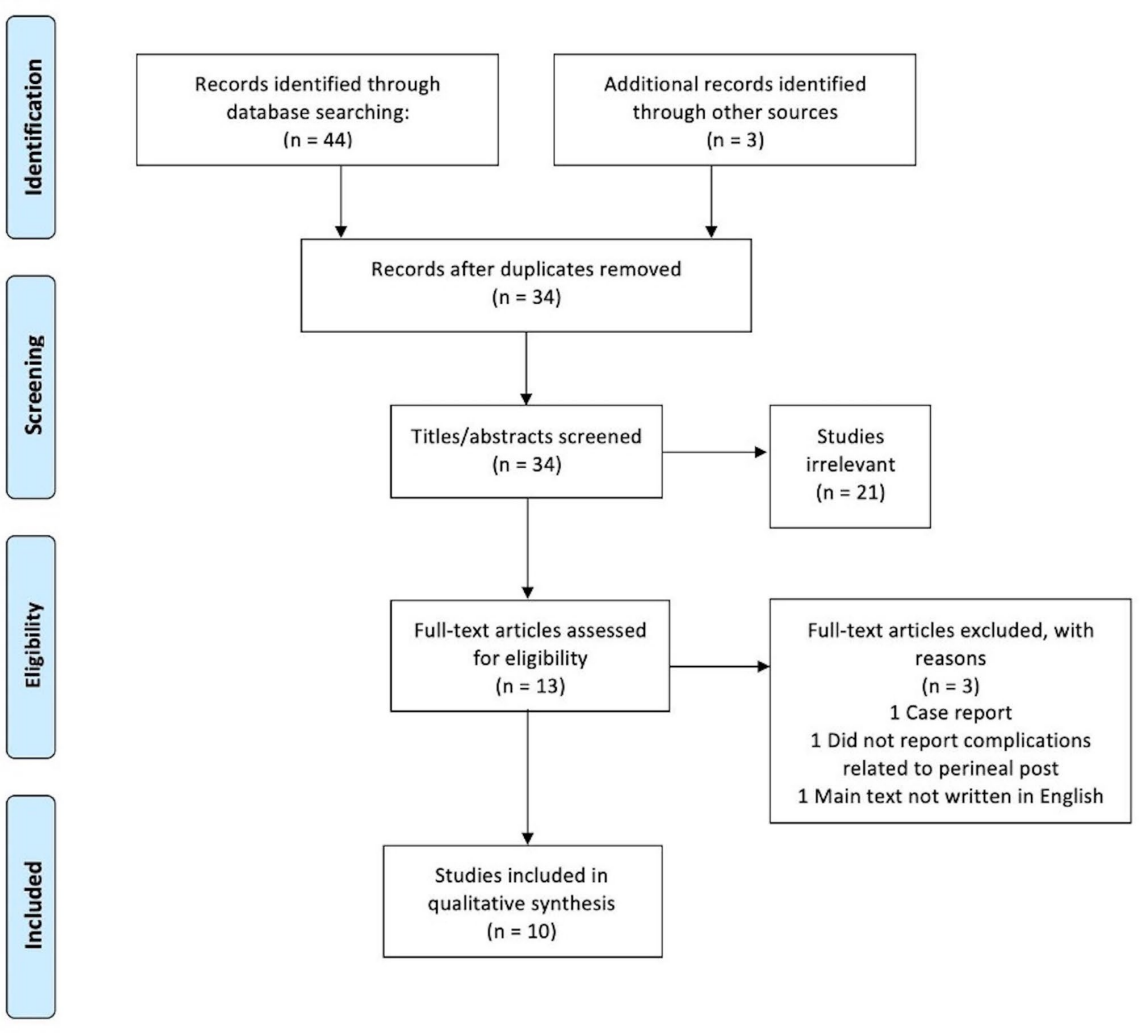
Flow diagram of study selection using PRISMA guidelines

The database search returned a total of 44 studies; 13 studies were identified as duplicates and 3 additional articles identified from additional sources were added. A total of 34 studies were screened using PRISMA guidelines (Fig. 1). After screening titles and abstracts, 21 studies were removed. Therefore, the full text of 13 studies were screened and 3 were excluded according to the exclusion criteria, with 10 studies remaining for qualitative review and analysis.

### Study characteristics

Of the 10 studies eligible, 2 (20%) were level III evidence,[15, 18] and 8 (80%) were level IV evidence (Table 3) [15, 17–20, 27–29]. A total of 351 patients who underwent femur fracture fixation were available from the 10 studies. Nine out of 10 studies (90%) [15, 17–20, 27–30] reported proportion of males from which the range of mean male percentage was 0–100%. Specific details on the femur fracture injury characteristics can be found in (Table 4). Mean age at surgery was 42.5 ± 14.9 years.

**Table 3:** Summary of Studies Included for Review

First Author (Year)	Level of Evidence	Study Design	Type of Study	Study Participants, n
Aprato et al. (2021)	3	Cohort comparison	Retrospective	95
Brumback et al. (1992)	4	Case series	Prospective	106
Coelho et al. (2008)	4	Case series	Retrospective	6
Hofmann et al. (1982)	4	Case series	Retrospective	4
Kao et al. (1993)	4	Case series	Retrospective	63
Mallet et al. (2005)	3	Cohort comparison	Retrospective	37
Parulekar et al. (2021)	4	Case series	Retrospective	3
Peterson et al. (1985)	4	Case series	Retrospective	4
Rajbabu et al. (2007)	4	Case series	Retrospective	4
Rose et al. (2007)	4	Case series	Prospective	29

**Table 4:** Demographic Information of Study Participants

First Author (Year)	Study Participants, n	Percent Male	Mean Age at Surgery, y	Mean Time from Injury to Surgery, days	Mechanism of Injury, n(%)	Injury Characteristics
Aprato et al. (2021)	95	–	***50.6 ± 18.1 (IQR: 38–62)	1.7; SD not reported	–	Uni-lateral femoral shaft fracture = 42(44%), subtrochanteric fractures = 53(56%); AO/OTA32A = 20(21%); AO/OTA32B = 18(19%); AO/OTA32C(16%); AO/OTA31A3 = 42(44%)
Brumback et al. (1992)	106	68.0%	–	–	–	Uni-lateral femoral shaft fracture = 106(100%)
Coelho et al. (2008)	6	100.0%	25.2 ± 3.2	–	MVA = 6(100%)	Uni-lateral diaphysial femoral fracture = 5(83%); Bi-lateral diaphysial femoral fracture = 1(17%)
Hofmann et al. (1982)	4	100%%	43.5 ± 12.8	–	–	Intertrochanteric fracture of the hip = 1(25%), subcapital fracture of the hip = 3(75%)
Kao et al. (1993)	63	66.7%	–	*3.7 ± 3.9	MVA = 42(66%); High Fall = 12(19%); Pathologic Fracture = 3(5%); Miscellaneous Trauma = 6(10%)	Uni-lateral femoral shaft fracture = 63(100%)
Mallet et al. (2005)	37	100.0%	27.1 ± 10.4	–	–	Uni-lateral femoral shaft fracture = 37(100%); AO/ASIF classification A = 22(59%); AO/ASIF classification B = 3(8%); AO/ASIF classification C = 12(32%)
Parulekar et al. (2021)	3	0.0%	33.3 ± 23.1	**2	MVA = 2(67%); Fall = 1(33%)	Uni-lateral femoral shaft fracture = 2(67%); intertrochanteric fracture = 1(33%); associated head and neck injury = 24(65%); associated spine injury = 5(14%); associated chest injury = 9(24%); associated pelvic injury = 6(16%); associated upper limb injury = 5(14%)
Peterson et al. (1985)	4	100.0%	24.3 ± 5.9	3.25 ± 2.2	MVA = 3(75%); Gunshot = 1(25%)	Angulated & displaced femoral shaft fracture = 1(25%); proximal femoral shaft fracture = 1(25%); comminuted distal femoral shaft fracture = 1(25%); femoral fracture with tibial-fibular fractures = 1(25%)
Rajbabu et al. (2007)	4	100.0%	27.0 ± 7.7	–	MVA = 4(100%)	Femur fracture + humerus fracture + laceration wounds over soft tissues of face = 1(25%); uni-lateral femoral shaft fracture = 2(50%); uni-lateral severely comminuted femoral shaft fracture = 1(25%)
Rose et al. (2007)	29	72%	***40.0 ± 14.1	–	MVA = 17(59%); Gunshot = 8(28%); Fall = 4(14%)	Femoral shaft fracture = 29(100%)
**Descriptive Statistics**	Total = 351	Range = 0–100%	Mean Range = 24.3–52.0	Mean Range = 1.7–3.7	MVA = 74 High Fall = 12 Fall = 4 Pathologic Fracture = 3 Gunshot = 9 Miscellaneous Trauma = 6	Femoral shaft fracture = 293 (83.5%) Subtrochanteric fracture = 53 (15.1%) Intertrochanteric fracture = 2 (0.6%) Subcapital fracture = 3 (0.9%)

“–“ denotes articles that did not report the variable; *sub-stratified by with and without pudendal nerve palsy; **only reported time to surg in 1/3 patients; “MVA” refers to Motor vehicle accident; ***Mean ± SD was estimated with IQR using methods reported by Luo et al. [44] and Wan et al. [45]

### Operative data

The mean operating time was reported by 7 studies (70%) [15, 17, 19, 20, 28, 29, 31] with a mean range of 1.7–3.7 h. Of the 351 patients, 324 (92.3%) were operated on in the supine position, 14 (4.0%) in the lateral decubitus position, 2 (0.6%) in the prone position, and the position of the patient was unreported for 11 (3.1%) patients. Two (0.6%) patients were treated with a modified Hagie pin fixation, 1 (0.25%) with in-situ pinning, 1 (0.25%) with sliding hip screw, and the remaining 347 patients were treated with intramedullary nailing (Table 5).

**Table 5:** Operative Information of Patients Treated on Traction Table with Perineal Post

First Author (Year)	Study Participants, n	Mean Operating Time, hours	Patient Position, n(%)	Fracture Fixation Method	Fracture Table Details	Countertraction Post Details	Perineal Post Dimensions
Aprato et al. (2021)	95	1.22, SD not reported	Supine = 95(100%)	Femoral shaft fractures treated with Trigen femoral nail (Smith&Nephew); Subtrochanteric fractues fixed proximally with 2 cephalic screws	–	–	–
Brumback et al. (1992)	106	*2.8 ± 0.6	Supine = 106(100%)	1st generation static interlocking fixation for 97 patients (92%); 2nd generation (reconstruction) static interlocking nailing for 9 patients (8%)	–	Maquet Orthostar (Simens Medical Systems, Iseline, NJ, USA)	Perineal post diameter = 4.1 cm; perineal post with rubber cylinder padding diamter = 6.8 cm
Coelho et al. (2008)	6	5.6 ± 2.1	–	Locked intramedullary antegrade nail fixation = 6 patients (100%)	–	–	–
Hofmann et al. (1982)	4	3.6 ± 1.1	Prone = 2(50%); Supine = 2(50%)	Muscle-pedicle graft and modified Hagie-pin fixation of the hip = 2(50%); Intramedullary nailing = 1(25%); In situ pinning of fracture = 1(25%)	–	–	–
Kao et al. (1993)	63	3.45 ± 1.19	Supine = 51(81%), lateral decubitus = 12(19%)	6 types of intramedullary nails used depending on availability/surgeon preference: (1) Brooker-Wills IM nail (Biomet, Warsaw, IN, USA); (2) Russell-Taylor femoral nail (Richards, Memphis, TN, USA); (3) Russell-Taylor Recon nail (Richards, Memphis, TN,USA); (4) Pathfinder nail (Biomet, Warsaw, In, USA); (5) Grosse-Kempf nail (Howmedica, Rutherford, NJ, USA); (6) Kuntscher nail (Howmedica, Rutherford, NJ, USA)	Amsco Orthographics 2 fracture table (American Sterilizer, Erie, PA, USA) for 44 patients (70%); Chick fracture table (Chick Medical Products, Greenwood, SC, USA) for 19 patients (30%)	Information found in “Fracture Table Details” column	Amsco Orthographics 2 fracture table = 3.5 cm diameter; (supine position)wrapped with cotton-cast padding/silicone roll = 6 cm diameter; (lateral decubitus position) wrapped with 3 layers of cotton-cast padding = 8 cm diameter Chick fracture table = 5.0 cm diameter; (supine position) wrapped with cotton-cast padding/silicone roll = 6 cm diameter
Mallet et al. (2005)	37	–	Supine = 37(100%)	Intramedullary nailing for femoral shaft fractures for 37 patients (100%)	Alphamaquet 1150 orthopedic table (Maquet, Getinge Surgical Systems, Getinge, Sweden)	Information found in “Fracture Table Details” column	–
Parulekar et al. (2021)	3	–	Unknown position = 3(100%)	Intramedullary nailing for femoral shaft fractures for 2 patients (67%); Sliding hip screw fixation for intertrochanteric fracture for 1 patient (33%)	–	–	–
Peterson et al. (1985)	4	3.80 ± 2.84	Supine = 2(50%), lateral decubitus = 2(50%)	Intramedullary nailing for femoral shaft fractures for 4 patients (100%)	–	–	–
Rajbabu et al. (2007)	4	**4.67 ± 1.15	Supine = 2(50%), unknown position = 2(50%)	Orthofix intramedullary nail for 3 patients (75%); Unknown treatment for 1 patient (25%)	–	–	–
Rose et al. (2007)	29	–	Supine = 29(100%)	Static Intramedullary nailing = 29(100%)	–	–	perineal post diameter = 3.8 cm; wrapped with cast padding diameter = 8 cm
**Descriptive Statistics**	Total = 351	Mean Range = 1.22–5.6	Supine = 324(92.3%) Lateral decubitus = 14(4.0%) Prone = 2(0.6%) Unknown = 11(3.1%)	Intramedullary nail = 347 (98.9%) Muscle-pedicle graft and modified Hagie-pin fixation of hip = 2 (0.6%) In-situ pinning of fracture = 1 (0.25%) Sliding hip screw = 1(0.25%)			

“–“ denotes articles that did not report the variable; *SD was estimated with IQR using methods reported by Wan et al. [45]; **Missing Operating Time for 1 patient

### Postoperative outcomes

Eight out of 10 articles (80%) reported pudendal nerve palsy in patients treated on a fracture table with perineal post [15, 17–19, 28, 29, 31]. The mean pudendal nerve palsy rate ranged 0–100% and the mean duration of symptoms was 10–639 days. Specific postoperative complications are noted in Table 6; erectile dysfunction was the most common complication relating to the perineal post reported in 35 patients (10.0%), unilateral sensory disturbance of labia, scrotum, or penis was the second-most common complication reported in 22 patients (6.3%), and perineal skin necrosis was the third-most common complication reported in 11 patients (3.1%). Erectile dysfunction was treated using phosphodiesterase-5 inhibitors and all cases resolved with the longest duration reported to be 2 years in a patient with prolonged traction of 4 h due to difficulties encountered during the procedure [28]. All patients that developed perineal skin necrosis healed through secondary intention. No permanent complications relating to pudendal neurapraxia or soft tissue injuries were reported at final follow-up timepoints. PROs were reported in one study [30] using the IIEF. The authors compared IIEF scores of patients who underwent femoral fracture fixation versus tibial shaft fracture fixation on the fracture table using perineal post and found lower mean scores in femur fracture patients for erectile function, orgasmic function, intercourse satisfaction and overall satisfaction aspects, whereas, mean sexual desire scores showed no statistical difference.

**Table 6:** Postoperative Clinical and Patient Reported Outcomes

First Author (Year)	Study Participants, n	Pudendal Nerve Palsy Rate, %	Duration of Pudendal Nerve Palsy	Complication Reported	International Index of Erectile Function
Aprato et al. (2021)	95	15/95 (16%)	Mean = 10 days, SD not reported	Failure of fixation = 1patient; ED = 2 patients	–
Brumback et al. (1992)	106	10/106 (9%)	Mean = 4 weeks (Range 1–11weeks) for 9/10 patients; Continued symptoms of altered sensation in penis and scrotum at 6 month follow-up for 1 patient	Unilateral sensory disturbance of labia, scrotum or penis = 10patients; ED = 1patient	–
Coelho et al. (2008)	6	0/6 (0%)	–	Cutaneous perineal necrosis involving the scrotal base = 6patients	–
Hofmann et al. (1982)	4	4/4(100%)	Mean = 250days ± 204days	Sensory disturbance of shaft and bulb of penis = 2 patients; sensory disturbance of right side of scrotum and anus = 1patient; sensory disturbance of right side of penis and right side of scrotum = 1 patient; ED = 4 patients	
Kao et al. (1993)	63	10/63 (16%)	Mean = 33.8days ± 55.0days	Unilateral sensory disturbance of labia, scrotum or penis = 10patients; peroneal palsy = 1patient; ED = 3patients; prolonged drainage = 1patient	–
Mallet et al. (2005)	37	15/37 (41%)	–	ED = 15patients	Erectile function (Score range: 1–30) = 23.1 ± 6.7 Orgasmic function(Score range: 0–10) = 7.9 ± 2.7 Sexual desire(Score range: 2–10) = 7.6 ± 1.4 Intercourse satisfaction(Score range: 0–15) = 10.3 ± 3.1 Overall satisfaction(Score range: 2–10) = 7.1 ± 2.6
Parulekar et al. (2021)	3	0/3 (0%)	–	Bilateral vulvar necrosis = 2patients; Unilateral vulvar necrosis = 1patient	–
Peterson et al. (1985)	4	3/4 (75%)	Mean = 84.67days ± 120.82days	Testicular swelling and scrotal edema = 2patients; cutaneous perineal necrosis = 2patients; ED = 2patients; Urinary retention = 1patient	–
Rajbabu et al. (2007)	4	4/4 (100%)	Mean = 639days ± 183 days	ED = 4patients	–
Rose et al. (2007)	29	8/29 (27.6%)	Mean = 28days ± 61.4days	Purely sensory disturbance of perineum = 4 patients; ED and sensory loss = 4patients	–
**Descriptive Statistics**	Total = 351	Mean Range = 0–100%	Mean Range = 10days–639days	Failure of fixation = 1 (0.2%) ED = 35 (10.0%) Unilateral sensory disturbance of labia, scrotum, or penis = 22 (6.0%) Peroneal palsy = 1 (0.2%) Prolonged drainage = 1 (0.2%) Cutaneous Perineal necrosis = 11 (3%) Testicular swelling and scrotal edema = 2 (0.4%) Urinary retention = 1 (0.2%)	

“–“ denotes articles that did not report the variable; “ED” denotes erectile dysfunction

### Appraisal of Quality of Study Methodology and Risk of Bias

The methodological quality and risk of bias for all studies and all comparative (scored out of 24) and non-comparative studies (scored out of 16) showed low mean global scores (16.7 ± 0.6 and 8.1 ± 1.5 respectively) thus correlating to high risk of bias.

## Discussion

The incidence of perineal post-related complications is a rare but devastating outcome with significant morbidity for patients following surgical intervention of femur fractures and other orthopaedic pathologies. To the best of our knowledge, this is the first study to systematically review the literature focusing on perineal post-related complications in the setting of femur fracture fixation. Due to the paucity of current literature evaluating this topic, there is a need to disseminate the findings of this study to increase awareness among orthopaedic traumatologists and to consider methods of avoiding such complications in the future.

A recent survey of surgeon preferences of operating table and patient positioning for midshaft femoral fracture intramedullary nailing found only 29% of surgeons in the USA who responded preferred to use a traction table compared to 89% of surgeons in Canada [9]. However, the survey had a 26% response rate and only included the mail-lists from AO North America to capture the surgeon practices in the USA. Therefore, the general trends in the use of traction table for surgical management of femur fracture remains unclear.

With the recent increasing popularity and expanded indications for hip arthroscopy [32, 33], there has been a plethora of literature regarding hip arthroscopy complications and outcomes [34]. Similar to a fracture table, most hip arthroscopy tables employ a padded perineal post in order to allow for adequate distraction of the hip joint and safe introduction of instrumentation [3, 35–37]. The most common complications reported within the literature following hip arthroscopy are related to the perineal post giving rise to pudendal, sciatic, and peroneal nerve neurapraxia [38]. With the perineal post being at the center of attention for causing the pudendal nerve-related complications, postless distraction techniques have been studied in hip arthroscopy and femoral nailing studies [31, 39–41]. In a prospective case series of 1,000 hip arthroscopy cases without a perineal post, Mei-Dan et al. [39] reported no pudendal nerve complications or soft tissue injuries to the perineum thereby demonstrating the efficacy of the specially designed distraction setup in combination with the Trendelenburg position. Aprato et al. [31] conducted a retrospective cohort comparison study of femoral shaft fractures treated with intramedullary nailing on a traction table with and without a perineal post. Two out of 95 patients treated on a table with a perineal post group reported pudendal nerve palsies, whereas none were reported in the postless group which included 50 patients and resulted in adequate distraction, reduction, and nailing of subtrochanteric and femoral shaft fractures. In both aforementioned studies, the Trendelenburg position was successfully used to create enough friction between the patient and the operating table to allow for distraction of the treated limb.

A recent systematic review by Wininger et al. [3] compared perineal post-related hip arthroscopy complications between 17 prospective studies and 74 retrospective studies which included 11,148 hips. The authors found that the incidence of post-related complications was 216/3032 (7.1%) in retrospective literature which was a five-fold increased incidence compared to 117/8116 (1.4%) in prospective hip arthroscopy literature. The incidence of pudendal nerve palsy may be higher than what is reported in hip arthroscopy literature due to longer duration of surgery and smaller perineal post dimensions. The perineal post with padding dimensions reported in this review ranged from 6 to 8 cm which is smaller than the recommendations made by Papavasiliou et al. [42] to use a well-padded post wide enough (diameter ≥ 9 cm) to distribute forces across a larger surface area. In Brumback et al’s prospective study [15], a strain-gauge was placed on the perineal post to detect the perineal pressure over the course of the surgery. The authors concluded that pudendal neurapraxia was correlated with the summated magnitude of intra-operative perineal pressure rather than the duration of the operation. Similar results have been shown in the hip arthroscopy literature. A recent study by Bailey et al. [4] concluded that postoperative pudendal nerve palsy is associated with the product of traction force and duration. Although the positioning of the patient on the surgical table may depend on several factors such as the specific model of table, fracture type and surgeon preference, further studies are needed to clarify whether the supine or lateral decubitus position places a greater risk for pudendal nerve-related complications [15, 17, 18, 43].

Our systematic review found that there is a lack of high-quality studies evaluating the complications related to the perineal post in femur fracture treatment. Future studies should aim to reduce or eliminate these complications with postless techniques as described by Aprato et al. [31]. Until further studies elucidate methods of reducing postoperative perineal nerve complications, surgeons must understand and appropriately convey the potential risks associated with use of a perineal post when engaging in preoperative discussions with patients. Furthermore, patients should be actively screened for any genitoperineal soft tissue complications and sensory disturbances postoperatively.

### Limitations

There are several limitations to this study. Most of the studies that were included were retrospective in nature and thus are subject to recall and confirmation bias leading to under-reporting or skewing of the complications being reported. Furthermore, the paucity of higher quality studies is revealed in the low mean MINORS scores for comparative and noncomparative studies, thereby demonstrating a high level of bias in the studies included. Eight of the included studies were published more than a decade ago; however, we included 2 papers published in 2021 which shows that perineal post-related complications still occur. Additionally, the follow-up time within the included studies were mostly unreported. Consequently, we are unable to provide meaningful long-term follow-up data. Finally, we were unable to provide a sub-group analysis of complications according to fracture type (femoral shaft, subtrochanteric, intertrochanteric, and subcapital fractures) as many studies did not report complication data sub-stratified by fracture type.

## Conclusion

The use of a perineal post when treating femur fractures on a fracture table poses risks for pudendal neurapraxia and perineal soft tissue injury. Post padding is mandatory and supplemental padding may also be required. Appropriate perineal skin examination prior to use is also important. Occurring at a higher rate than previously thought, appropriate post-operative examination for any genitoperineal soft tissue complications and sensory disturbances should not be ignored.

## Data Availability

The datasets used during the current study are available from the corresponding author on reasonable request.

## Declarations

### Author FAL reports

AAOS: Board or committee member. AO: Unpaid consultant. Biomet: IP royalties; Paid consultant; Paid presenter or speaker; Research support. DePuy, A Johnson & Johnson Company: IP royalties; Research support. Orthopaedic Trauma Association: Board or committee member. Stryker: IP royalties. Synthes: Paid consultant; Paid presenter or speaker.
